# Supplementary material for: Netcombin: An algorithm for constructing optimal phylogenetic network from rooted triplets
Source: PLoS One. 2020 Sep 18;15(9):e0227842. doi: 10.1371/journal.pone.0227842 (PMC7500971; doi:10.1371/journal.pone.0227842)
Supplement: S4 File — (ZIP) [file pone.0227842.s004.zip › SequenceData-Triplet/sequenceData-10-20-30-0-Triplet/length 300, numseq 20, rate 10^-7/10 seq.pdf]

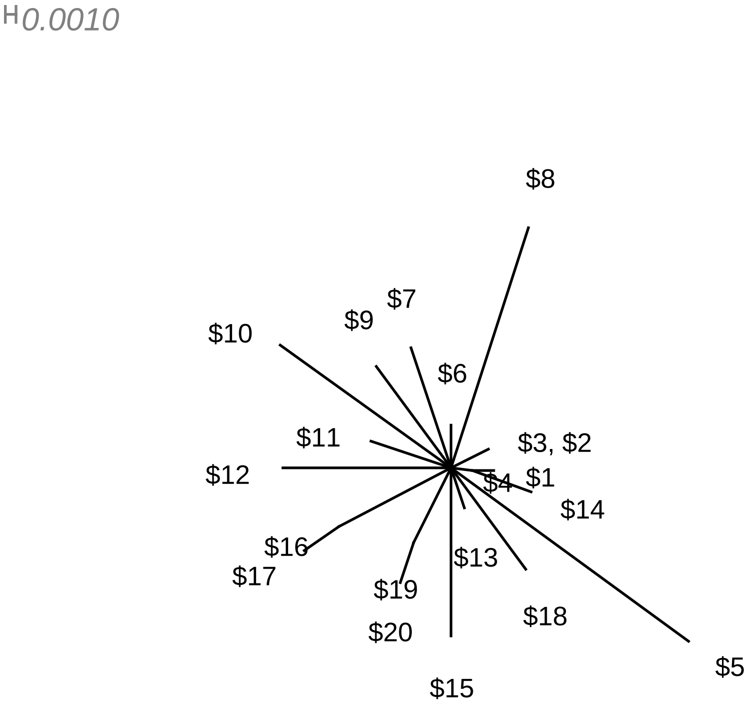
0.0010
$8
$7
$9
$10
$6
$11
$3, $2
$12
$1
$4
$14
$16
$13
$17
$19
$18
$20
$5
$15
